# Metabolite Profiling of Eastern Teaberry (*Gaultheria procumbens* L.) Lipophilic Leaf Extracts with Hyaluronidase and Lipoxygenase Inhibitory Activity

**DOI:** 10.3390/molecules22030412

**Published:** 2017-03-06

**Authors:** Piotr Michel, Aleksandra Owczarek, Magdalena Matczak, Martyna Kosno, Paweł Szymański, Elżbieta Mikiciuk-Olasik, Anna Kilanowicz, Wiktor Wesołowski, Monika A. Olszewska

**Affiliations:** 1 Department of Pharmacognosy, Faculty of Pharmacy, Medical University of Lodz, 1 Muszynskiego, 90-151 Lodz, Poland; aleksandra.owczarek@umed.lodz.pl (A.O.); magdalena.matczak@umed.lodz.pl (M.M.); kosno.martyna@gmail.com (M.K.); monika.olszewska@umed.lodz.pl (M.A.O.); 2 Department of Pharmaceutical Chemistry, Drug Analyses and Radiopharmacy, Medical University of Lodz, 1 Muszynskiego, 90-151 Lodz, Poland; pawel.szymanski@umed.lodz.pl (P.S.); elzbieta.mikiciuk-olasik@umed.lodz.pl (E.M.-O.); 3 Department of Toxicology, Faculty of Pharmacy, Medical University of Lodz, 1 Muszynskiego, 90-151 Lodz, Poland; anna.kilanowicz@umed.lodz.pl (A.K.); wiktor.wesolowski@umed.lodz.pl (W.W.)

**Keywords:** *Gaultheria procumbens*, leaves, lipophilic extracts, GC-MS, anti-inflammatory activity, seasonal variation

## Abstract

The phytochemical profile and anti-inflammatory activity of *Gaultheria procumbens* dry lipophilic leaf extracts were evaluated. Forty compounds were identified by GC-MS, representing 86.36% and 81.97% of the petroleum ether (PE) and chloroform (CHE) extracts, respectively, with ursolic acid (28.82%), oleanolic acid (10.11%), methyl benzoate (10.03%), and methyl salicylate (6.88%) dominating in CHE, and methyl benzoate (21.59%), docosane (18.86%), and octacosane (11.72%) prevailing in PE. Three components of CHE were fully identified after flash chromatography isolation and spectroscopic studies as (6*S*,9*R*)-vomifoliol (4.35%), 8-demethyl-latifolin (1.13%), and 8-demethylsideroxylin (2.25%). Hyaluronidase and lipoxygenase inhibitory activity was tested for CHE (IC_50_ = 282.15 ± 10.38 μg/mL and 899.97 ± 31.17 μg/mL, respectively), PE (IC_50_ = 401.82 ± 16.12 μg/mL and 738.49 ± 15.92 μg/mL), and nine of the main constituents versus heparin (IC_50_ = 366.24 ± 14.72 μg/mL) and indomethacin (IC_50_ = 92.60 ± 3.71 μg/mL) as positive controls. With the best activity/concentration relationships, ursolic and oleanolic acids were recommended as analytical markers for the extracts and plant material. Seasonal variation of both markers following foliar development was investigated by UHPLC-PDA. The highest levels of ursolic (5.36–5.87 mg/g DW of the leaves) and oleanolic (1.14–1.26 mg/g DW) acids were observed between August and October, indicating the optimal season for harvesting.

## 1. Introduction

*Gaultheria procumbens* L. (eastern teaberry, checkerberry, American wintergreen, Ericaceae) is a small, low-growing, evergreen shrub native to northeastern North America and widely cultivated all through the Northern Hemisphere due to its ornamental and medicinal value. The most commonly used plant organs of *G. procumbens* are the leaves, traditionally valued as anti-inflammatory and analgesic agents as well as a source of wintergreen essential oil [1]. Methyl salicylate, constituting over 95% of the essential oil [2], and its glycosidic precursor—gaultherin [3]—are the main representatives of salicylates, so far considered as the primary active constituents of *Gaultheria* plants [4,5,6]. However, plant extracts are usually multicomponent and their activity is rarely due to one, single constituent. In fact, our previous studies [7] demonstrated that the anti-inflammatory activity of wintergreen leaves is also largely influenced by other polar compounds, among which thirty-five representatives of flavonoids, proanthocyanidins, and caffeoylquinic acids were identified by UHPLC-PDA-ESI-MS^3^ profiling. The concept of metabolite profiling evolved recently in the most preferable characterization procedure of bioactive plant fractions, but it is generally confined to hydrophilic compounds alone [8], while lipophilic components are often studied only fragmentarily, either by classic techniques such as isolation, or after separation of less complex, specific sub-fractions, e.g., essential oils. That is also the case of *Gaultheria* plants, in which the presence of several lipophilic constituents, such as pentacyclic triterpenes and sterols, was discovered in selected taxons (*G. fragrantissima*, *G. nummularoides*, *G. paniculata*, *G. subcorymbosa*, and *G. yunnanensis*) by standard (isolation) techniques [1,9,10,11,12,13]. More selective methods (GC-MS) have so far been used only for identification of long-chain aliphatic alkanes C_15_–C_20_ in leaf waxy layers of *G. subcorymbosa* and *G. antipoda* [14], and volatile fractions of various wintergreen species [1,2]. Some of the isolated non-volatile molecules, including pentacyclic triterpene acids, like oleanolic and ursolic acids, isomeric triterpene alcohols, like α- and β-amyrins, as well as phytosterols with the ubiquitous β-sitosterol, are well-documented anti-inflammatory agents, active in certain in vitro and in vivo models [15,16,17,18,19,20]. Despite these facts, there is no comprehensive information available on the lipophilic profile of any *Gaultheria* plant and its possible impact on the anti-inflammatory activity of the respective plant materials. As qualitative and quantitative composition of specialized plant metabolites often depends strongly on numerous factors, among which stage of the phenological cycle is one of the most important [21,22], seasonal dynamics in biosynthesis of the primary lipophilic components could also significantly influence the final biological value of *Gaultheria* plants and their reasonable harvest. However, also this issue remains undiscovered for wintergreen species.

Thus, the aim of this research was to determine the phytochemical profile and anti-inflammatory activity of dry lipophilic (petroleum ether and chloroform) extracts of *G. procumbens* leaves. The qualitative profile of the extracts was first monitored by GC-MS. Next, the preparative liquid column chromatography, followed by spectroscopic (ESI-MS, UV-Vis, IR, 1D and 2D NMR) studies, was performed for full structural characterization of some problematic analytes. Two in vitro tests measuring the inhibitory effects on pro-inflammatory enzymes, i.e., lipoxygenase and hyaluronidase, were selected as anti-inflammatory activity models, based on the previous findings for dry hydrophilic leaf extracts of wintergreen [7]. Finally, the seasonal variations in the content of two dominant lipophilic biomolecules, i.e., ursolic and oleanolic acids, in the leaf samples of *G. procumbens* were monitored throughout the growing season by UHPLC-PDA.

## 2. Results and Discussion

### 2.1. Qualitative Profiling of G. procumbens Dry Lipophilic Leaf Extracts

The qualitative characteristics of the wintergreen leaf lipophilic compounds were evaluated by GC-MS analysis of the dry petroleum ether (**PE**) and chloroform (**CHE**) extracts after trimethylsilyl derivatization. The main identification data of all detected peaks and the representative GC chromatograms of **PE** and **CHE** are presented in Table 1 and Figures S1 and S2, respectively (for full data see Table S1).

The GC-MS analysis of **PE** revealed the presence of over sixty constituents, thirty-two of which, representing 86.36% of the analyzed extract, were identified by comparing their retention times and MS profiles with those of reference standards as well as with literature data and library databases. According to the spectroscopic profiles, four major groups could be distinguished among analytes, including aliphatic hydrocarbons (**8**, **12**, **13**, **15**, **17**, **20**, **22**, **24**, **27**, **34**), alcohols (**10**, **16**, **23**, **28**, **29**), and carboxylic acids (**5**, **9**, **11**, **14**, **19**, **33**), as well as terpenoids (**35**–**40**). The dominant group were waxy substances (47.72% of the total peak area observed in the extract) represented by simple aliphatic hydrocarbons, among which docosane (**27**; 18.86%) and octacosane (**22**; 11.72%) were present at the highest percentage, as well as by aliphatic alcohols and carboxylic acids. Waxy alcohols (1.39%) and fatty acids (2.34%) occurred in small amounts, and only *n*-hexadecanoic acid (palmitic acid; **9**) exceeded the threshold of one percent (1.42%). Terpenoids and simple phenols, which also constituted a significant proportion of the extract, were represented by methyl benzoate (**3**; 21.59%), ursolic acid (**40**; 4.27%), α-amyrin (**38**; 3.86%), β-sitosterol (**36**; 2.68%), oleanolic acid (**39**; 1.70%) and methyl salicylate (**1**; 2.31%).

Some waxy substances and terpenoic derivatives listed above were also identified in **CHE**. The GC-MS analysis of this extract revealed the presence of thirty two lipophilic constituents, among which fourteen, representing 81.97% of the extract, were fully identified. Comparing to **PE**, the group of simple aliphatic hydrocarbons (**8**, **21**, **25**; 6.40%), alcohols (**4**; 2.78%), and carboxylic acids (**6**, **9**; 5.79%) occurred in **CHE** at lower levels, and the primary constituents were ursolic acid (**40**; 28.82%), oleanolic acid (**39**; 10.11%), methyl benzoate (**3**; 10.03%), and methyl salicylate (**1**; 6.88%).

This study is the first to explore chemical composition of petroleum ether and chloroform extracts of *G. procumbens* leaves. Although the literature for other *Gaultheria* species concerning the lipophilic components is fragmentary, it is generally in accordance with our findings. For instance, the aliphatic alkanes, i.e., pentadecane, hexadecane, heptadecane, octadecane, nonadecane, eicosane, heneicosane, docosane, and tricosane, were identified by GC-MS as constituents of waxy layers on the *G. subcorymbosa* and *G. antipoda* leaf surface [14]. An aliphatic long chain alkane (*n*-dotriacontane) was identified in aerial parts [12], and palmitic acid in roots [1] of *G. yunnanensis*. Reports have also indicated the presence of squalene [13], octadecanol, and palmitic acid [1] in the whole plant of *G. nummularioides*, and palmitic and stearic acids in the whole plant of *G. itoana* [9]. Similarly, ursolic and oleanolic acids, commonly occurring in the Ericaceae family and identified in this study as the major components of the lipophilic leaf extracts of *G. procumbens*, were observed earlier in the leaves of *G. adenothrix*, *G. fragrantissima*, and *G. subcorymbosa* [1], roots of *G. yunnanensis* [1], and the whole plant of *G. nummularioides* [13] and *G. yunnanensis* [1,11,12].

**Figure 1:**
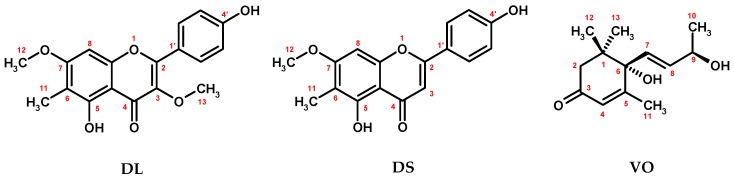
Structures of compounds **DL**, **DS** and **VO** isolated from *G. procumbens* chloroform extract.

The preliminary GC investigations of **CHE** revealed the presence of some further components that could not be identified by MS. Three of them occurred in the extract at the levels allowing preparative isolation. The compounds in question gave a purple fluorescence in UV and/or a positive reaction with Liebermann-Burchard reagent indicating phenolic or terpenoic structures. The subsequent preparative open column chromatographic separation followed by flash chromatography afforded pure compounds (Figure 1), NMR data of which are provided in Table 2 and Table 3.

Compounds **DL** and **DS** (Figure 1) were isolated as yellow needles. The ESI-MS spectra of both analytes recorded in the negative-ion mode exhibited [M − H]^−^ ions at *m*/*z* 327 and 297, respectively, which were consistent with the molecular weights of 328 and 298, and the molecular formulas C_18_H_16_O_6_ and C_17_H_14_O_5_, respectively. The UV-Vis spectra of compounds **DL** and **DS** with maxima at 270–275 and 330–340 nm as well as positive results of the Shinoda reaction with the orange color specific to flavone derivatives led to their classification as flavonoids.

The ^1^H- and ^13^C-NMR spectra of **DL** in CDCl_3_ (Table 2) revealed the presence of proton and carbon signals typical of a 3,5,6,7,4′-pentasubstituted flavone [23]. Specifically, signals of five aromatic protons found in the ^1^H-NMR at δ_H_ 7.97 (2H, d, *J* = 8.7 Hz), δ_H_ 6.89 (2H, *d*, *J* = 8.7 Hz), and δ_H_ 6.38 (1H, s) indicated a *para* substitution pattern of the B ring and the presence of an isolated proton on the A ring. Two three-proton singlets observed at δ_H_ 3.84 and δ_H_ 3.79 were assigned to methoxyl groups linked at C-3 and C-7 positions, respectively, according to the HMBC cross peaks with carbon resonances at δ_C_ 139.1 and δ_C_ 163.5, characteristic of a 3,7-di-*O*-substituted flavone. This substitution pattern was further confirmed by UV-Vis analysis with complexation reagents [24]. The third three-proton singlet found in the ^1^H-NMR at δ_H_ 2.05 was attributed to a methyl group at the C-6 position by HMBC cross peaks with the characteristic C-7 and C-5 (δ_C_ 158.3) carbon resonances. The substitution position of the single A-ring proton was next assigned to C-8 according to the HMBC correlations with the C-7 and C-9 (δ_C_ 155.0) resonances. The recorded spectral data, consistent with the literature [25,26], led to the identification of compound **DL** as 4′,5-dihydroxy-3,7-dimethoxy-6-methylflavone (8-demethyllatifolin).

The ^1^H- and ^13^C-NMR spectra of **DS** in CDCl_3_ (Table 2) were similar to those of compound **DL**, with one exception. Specifically, the three-proton signal of the methoxyl group at C-3 position was replaced by a one-proton singlet at δ_H_ 6.58 correlating with the carbon signal at δ_C_ 101.1 in the HMQC spectrum, which was, nevertheless, still assigned to C-3 position due to the observed large upfield shift (Δδ_C_ = −38.0) of the carbon resonance after cleavage of an electronegative (methoxyl) substituent. Eventually, according to the recorded and literature data [26,27] compound **DS** was identified as 4′,5-dihydroxy-7-methoxy-6-methylflavone (8-demethylsideroxylin).

Compound **VO** (Figure 1) was isolated as white needles. The ESI-MS spectrum recorded in the positive-ion mode exhibited the presence of pseudomolecular [M + H]^+^ ion at *m*/*z* 225, which was consistent with the molecular weight of 224 and molecular formula C_13_H_20_O_3_. The tested compound gave a positive reaction with Liebermann-Burchard reagent, yielding the yellow color specific to low-molecular terpenoic derivatives. The ^1^H-NMR spectrum of **VO** in CDCl_3_ (Table 3) showed the presence of proton signals characteristic of a substituted α-ionol [28], i.e., three olefinic proton signals at δ_H_ 5.90 (1H, br s), 5.85 (1H, dd, *J*_1_ = 5.3 Hz, *J*_2_ = 15.8 Hz), and 5.79 (1H, dd *J*_1_ = 0.8 Hz, *J*_2_ = 15.8 Hz), as well as four resonances of methyl groups, including two doublets at δ_H_ 1.89 (3H, d, *J* = 1.1 Hz) and 1.30 (3H, d, *J* = 6.4 Hz), and two singlets at δ_H_ 1.08 and 1.01. Out of the five remaining proton signals typical of α-ionol, only two resonances, with the geminal coupling constant at δ_H_ 2.44 ppm (1H, d, *J* = 16.9 Hz) and 2.24 ppm (1H, d, *J* = 16.9 Hz), were present, indicating substitution at the cyclohexane ring. In the IR spectrum of **VO** signals of the carbonyl (C=O) and hydroxyl groups (OH) were visible at 1659 cm^−1^ and 3355 cm^−1^, respectively. The position of the carbonyl group was assigned to C-3 based on the HMBC cross peak between the olefinic proton signal at C-4 (δ_H_ 5.90, 1H, br s) and the carbonyl resonance (δ_C_ 197.9). The position of the hydroxyl group at C-6 was next determined according to the large downfield shift (Δδ_C_ = +24.7) for C-6 carbon signal in comparison with the predicted corresponding signal of α-ionol [28]. The postulated chemical structure was confirmed by ^13^C-NMR and 2D NMR (^1^H-^1^H COSY, HMQC, HMBC) experiments. The absolute configuration of the chiral centers in the compound **VO** was determined by measuring the optical rotation ${\left[\alpha \right]}_{D}^{20}$ = +211.5° (*c* = 1.06 g/100 mL, CHCl_3_), that stands in accordance with the literature data for the (6*S*,9*R*) isomer [29]. Finally, the compound **VO** was identified as (6*S*,9*R*)-vomifoliol, that is (6*S*,9*R*)-6-hydroxy-3-oxo-α-ionol.

This work is the first report on the presence of (6*S*,9*R*)-vomifoliol (**VO**) in the leaves of *G. procumbens* and in the whole genus *Gaultheria*. Vomifoliol occurs commonly in the plant kingdom, but was earlier identified in Ericaceae family only by GC-MS as the component of the aroma fraction isolated from *Arbutus unedo* honey [30]. Two rare lipophilic flavonoid aglycones, i.e., 8-demethyllatifolin (**DL**) and 8-demethylosideroxylin (**DS**), were reported previously as components of the lipophilic wax layer on the *G. procumbens* leaf surface but with structure analysis based only on UV-Vis spectroscopy and lacking NMR data [26]. Apart from *G. procumbens,* compound **DL** was also identified in a few representatives of Pinaceae and Myrtaceae, and **DS** in those of Ranunculaceae, Hypericaceae, and Myrtaceae [25,27,31].

### 2.2. Activity of G. procumbens Dry Lipophilic Leaf Extracts on Two Enzymes Involved in Inflammation

Inflammation is a part of the complex biological response of body tissues to harmful stimuli, such as pathogens or irritants. During the process, different cell types like macrophages and monocytes are recruited, what leads to the regulated production of various pro- and anti-inflammatory mediators, including cytokines, chemokines, and inducible enzymes [32]. Among many methods used for activity screening of anti-inflammatory agents, one of the most commonly employed techniques is based upon the ability of natural compounds to inhibit the pro-inflammatory enzymes, which include *i.a.* lipoxygenases and hyaluronidase.

Therefore, the inhibitory effects of lipophilic extracts of the wintergreen leaves and their pure constituents representing all metabolite groups found by GC-MS towards enzymes from both aforementioned pro-inflammatory classes were measured in this study.

**Figure 2:**
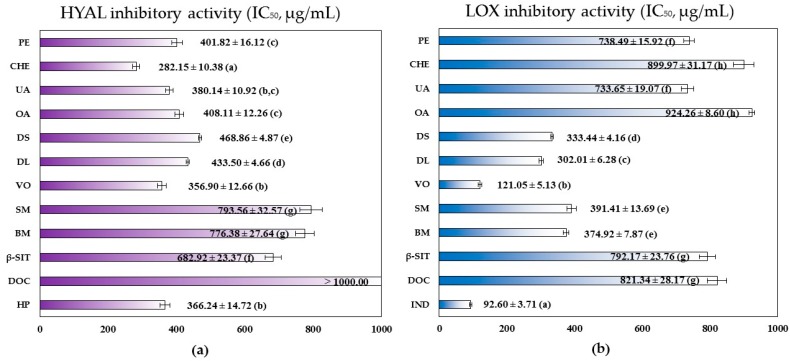
Anti-inflammatory activity of *G. procumbens* dry lipophilic leaf extracts; inhibitory activity on: (**a**) hyaluronidase and (**b**) lipoxygenase. Results are presented as mean values ± SD (*n* = 3 × 2) represented by error bars; IC_50_, 50% inhibition of enzyme activity; for each parameter different lowercase letters given in parentheses (a–h) indicate significant differences between the mean values (*p* < 0.05) in the Tukey’s test; abbreviations: **PE**, petroleum ether extract; **CHE**; chloroform extract; **UA**, ursolic acid; **OA**, oleanolic acid; **DS**, 8-demethylsideroxylin; **DL**, 8-demethyllatifolin; **VO**, (6*S*,9*R*)-vomifoliol; **SM**, methyl salicylate; **BM**, methyl benzoate; **β-SIT**, β-sitosterol; **DOC**, docosane; positive standards: **IND**, indomethacin; **HP**, heparin.

As presented in Figure 2, the strongest inhibitory potential towards hyaluronidase was demonstrated by **CHE** (IC_50_ = 282.15 ± 10.38 μg/mL), which exhibited 1.3 times higher inhibitory activity than positive heparin control (IC_50_ = 366.24 ± 14.72 μg/mL) and 1.4 times stronger capacity than **PE** (IC_50_ = 401.82 ± 16.12 μg/mL). The anti-hyaluronidase activity of **CHE** and **PE** was comparable or even higher than that of the most active terpenoid and flavonoid components of the extracts, such as **UA**, **OA**, **DS**, **DL** and **VO**. The activity of **SM**, **BM** and **β-SIT** was, in turn, much weaker—2.4–2.8 times lower than that of **CHE**, and 1.7–2.0 times lower than that of **PE**. At the same conditions, **DOC** presented no inhibitory activity towards hyaluronidase in the tested concentration range. Comparison of the activity parameters and GC peak areas observed for all analytes indicated, on one hand, significant synergistic effects of components of both extracts, and the dominant impact of **UA** and **OA** on the anti-inflammatory activity of **CHE** (according to their high abundance of 28.82% and 10.11% of the extract, respectively) on the other.

The analyzed lipophilic extracts and their constituents were in general less effective inhibitors of lipoxygenase than hyaluronidase (Figure 2). Among the extracts, higher inhibitory activity towards lipoxygenase was this time observed for **PE** (IC_50_ = 738.49 ± 15.92 μg/mL), which was, nevertheless, 8 times lower than the activity of positive indomethacin control (IC_50_ = 92.60 ± 3.71 μg/mL). The inhibitory potential of **CHE** (IC_50_ = 899.97 ± 31.17 μg/mL) was, in turn, about 9.7-fold lower than in the case of indomethacin. The anti-lipoxygenase activity of **CHE** and **PE** was comparable to that of **UA**, **OA**, **β-SIT** and **DOC**. Other components of the extracts, i.e., **DS**, **DL**, **SM** and **BM**, were 2.3–3.0 times stronger inhibitors of lipoxygenase than **CHE**, and 1.9–2.4 times more effective than **PE**, but still noticeably less active (3–4 times weaker) than the positive control. Only the low-molecular terpenoic derivative (**VO**) presented activity (IC_50_ = 121.97 ± 5.13 μg/mL) comparable to that of indomethacin, which suggested possible significant impact of **VO** on the anti-inflammatory activity of the wintergreen leaves. On the other hand, **VO** was present just in the less active extract (**CHE**), and only at the low concentration of 4.35%. Similar observations can be made for three further most active analytes, i.e., **SM**, and both flavonoids (**DL** and **DS**), which were absent from the more effective extract (**PE**) or found only at low levels. Therefore none of the tested in the present study components could be indicated as solely responsible for the anti-lipoxygenase activity of the extracts, as it corresponded rather to additive and/or synergistic effects of all analytes. Nevertheless, the relatively high contents of **BM** (21.59%) and **DOC** (18.86%) in **PE**, and **UA** (28.82%), **OA** (10.11%), and **BM** (10.03%) in **CHE** may indicate their considerable relevance to the inhibitory activity towards lipoxygenase.

To the best of our knowledge, this work is the first to compare in one study design the hyaluronidase and lipoxygenase inhibitory activity of natural lipophilic compounds representing various chemical groups, such as salicylates, phytosterols, triterpene acids, C_13_-norisoprenoids, aliphatic alkanes, and methoxylated flavonoids, and therefore provides better insight into the nature and extent of the activity of lipophilic plant extracts. The work is also the first to provide the target activity parameters for **DS**, **DL**, **VO** and **DOC**.

As indicated above, the anti-inflammatory activity of the analyzed extracts was due to additive and/or synergistic effects of their numerous components. However, as especially high inhibitory effect was observed towards hyaluronidase, the major determinants of this activity, i.e., **UA** and **OA**, should be recommended as analytical markers for standardization of the *G. procumbens* extracts and raw leaf material.

Triterpene compounds, represented in the wintergreen extracts mainly by **UA** and **OA**, are widely distributed in the plant kingdom and characterized by a vast range of pharmacological activities [17,33]. Triterpene acids exhibit the ability to reduce inflammation in various mechanisms, e.g., by inhibition of enzymes involved in the inflammatory response. Apart from hyaluronidase and lipoxygenase analyzed in the present study, they may also influence phospholipase A2, cyclooxygenase (COX), nitric oxide synthase, and elastase [34]. Moreover, the acids can reduce the formation of pro-inflammatory mediators, such as prostaglandins and cytokines [35]. The ability of **UA** to attenuate expression of COX-2 and the secretion of pro-inflammatory cytokines, like tumor necrosis factor (TNF-α), interferon γ (IFN-γ) and interleukins (e.g., IL-6), is also associated with its anticancer activity since chronic inflammation is recognized as a cancerogenesis-promoting condition [36]. The activity profile of the aforementioned triterpene acids suggests that for **CHE**, proven in the present work to be a good source of **UA** (28.82%) and **OA** (10.11%), a wide range of possible applications as an anti-inflammatory agent could be expected.

The dominant components of **PE** were aromatic esters—**BM** (21.59%) and **SM** (2.31%)—and waxy substances represented mainly by **DOC** (18.86%) and octacosane (11.72%). While natural salicylates have well documented anti-inflammatory activity [4,5,6], the potential of waxy substances is described only indirectly by activity profiling of wax-rich plants. According to the literature, significant amounts of simple aliphatic hydrocarbons, fatty alcohols (*n*-docosanol, *n*-octacosanol), saturated (lauric and palmitic acids) and unsaturated fatty acids (oleic, linoleic and linolenic acids), which are common components of waxy layers coating aerial organs of plants, are present in lipophilic extracts of some valuable herbal materials, such as *Prunus africana* bark, *Serenoa repens* fruits, and *Cucurbita pepo* seeds [37,38,39,40,41]. The waxy constituents of these plant materials are co-responsible, together with the fraction of phytosterols, for alleviating the inflammation-related symptoms of some urinary disorders such as benign prostatic hyperplasia via modulation of pro-inflammatory genes, inhibition of COX synthesis, and alteration in NF-κB signal transduction pathways [42]. Free fatty acids can act as anti-inflammatory agents also by reducing activation of leukocytes and decreasing the production of reactive oxygen species and pro-inflammatory mediators, like IL-1β and TNF-α [43,44]. Taking into account the biological activity of waxy substances, the existing literature indicates potentially broad application of wintergreen **PE**, especially rich in this group of components. The *G. procumbens* extract is also characterized by high extraction yield (12.2%), and thus it could be considered as an interesting alternative to the commercially available products.

### 2.3. Seasonal Variation in the Content of Triterpene Acids in G. procumbens Leaves

The triterpene acids **UA** and **OA**, recommended as analytical markers of anti-inflammatory active wintergreen lipophilic extracts, were simultaneously determined by UHPLC-PDA in the leaf samples of *G. procumbens* harvested monthly across the full growing season. The validated assay protocol, optimized to assure the best recovery of both analytes from plant materials, was previously developed in our laboratory [45] for standardization of Ericaceae herbal medicines.

**Figure 3:**
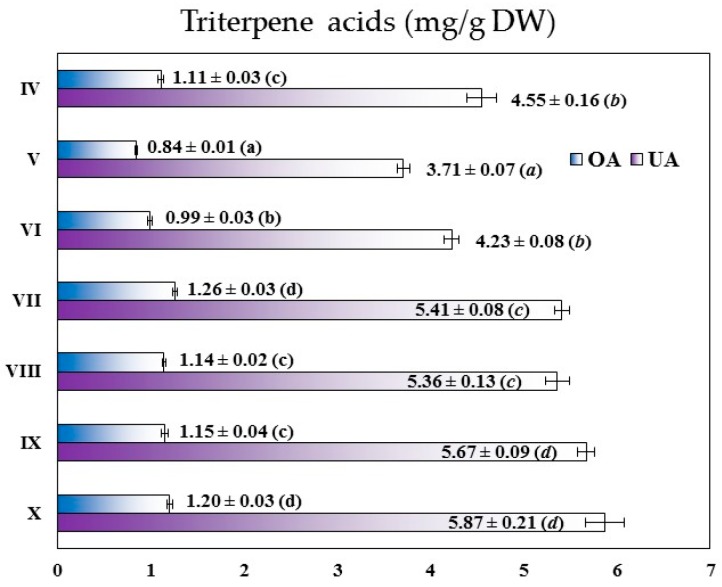
Seasonal variation (IV-X 2014) in the content of oleanolic and ursolic acid in the leaves of *G. procumbens*. Results are presented as mean values calculated per dry weight of the plant material ± SD (*n* = 3 × 3) represented by error bars, different superscripts (a–d) indicate significant differences in the mean values at *p* < 0.05 by the Tukey’s test; abbreviations: **OA**, oleanolic acid; **UA**, ursolic acid.

In all of the tested plant samples, **OA** and **UA** constituted the dominating part of the triterpene acid fraction, with other triterpene peaks negligible or not existing at the detection wavelength of 215 nm (Figure S3). The content of **UA** and **OA** in the investigated plant samples varied from 3.71 ± 0.07 to 5.87 ± 0.21 mg/g DW and 0.84 ± 0.01 to 1.26 ± 0.03 mg/g DW of the plant material, respectively (Figure 3). With the levels generally 4.5 times higher than those of **OA**, and reaching approximately 81.5%–83.1% of the total triterpene acid content, the primary constituent in all samples was **UA**. The highest concentrations of **UA** (5.67–5.87 mg/g DW) were found for two autumn months (September–October) followed by the months of late summer (July–August; 5.36–5.41 mg/g DW). In spring and early summer (April–June) the **UA** levels were significantly lower (*p* < 0.05) and the differences were up to 37% of the highest October concentration. The variations in **OA** levels were less pronounced (0.84–1.26 mg/g DW), and the peak contents were observed in July and October (1.20–1.26 mg/g DW). However, in early spring (April), late summer (August), and early autumn (September), the tested leaves accumulated only slightly lower **OA** levels (1.11–1.15 mg/g DW). That means that high amounts of the triterpene acids were biosynthesized in a relatively long vegetation period, i.e., between August and October (6.50–7.07 mg/g DW), indicating potentially good elasticity when selecting the harvest time. As similar seasonal dynamics was found earlier for polar anti-inflammatory constituents of *G. procumbens* leaves [21], the plant could be considered attractive for industrial purposes and affording both high quality lipophilic and polar extracts at the same, relatively wide vegetation period.

Free triterpene acids **UA** and **OA** occur commonly in different species of the Ericaceae family. One of the plant materials richest in these compounds are bearberry leaves (*Arctostaphylos uva-ursi*), which may contain up to 15 mg/g DW of the sum of triterpene acids, including 12.4 mg/g DW of **UA** and 2.7 mg/g DW of **OA** [46]. The triterpene acids content in other Ericaceae plants is generally lower. For instance, the leaves of lingonberry (*Vaccinium vitis-idaea*) contained 2.50–6.76 mg/g DW of **UA** and 0.74–2.15 mg/g DW of **OA** [45,47,48], while the content of both acids in the bilberry leaves (*Vaccinium myrtillus*) varied in the range of 0.75–1.30 mg/g DW for **UA** and 0.28–1.78 mg/g DW for **OA** [45,49,50]. Similarly, the sum of **UA** and **OA** in the leaves of *Rhododendron adamsii* reached 3.1 mg/g DW [51]. Taking into account the literature, the **UA** and **OA** levels observed in the present study put *G. procumbens* in a very good position comparing to other Ericaceae members, and the analyzed leaves could be thus considered good source of the isomeric triterpene acids.

## 3. Materials and Methods

### 3.1. General Information

HPLC grade reagents and standards, such as bovine testis hyaluronidase; bovine serum albumin; hyaluronic acid; lipoxygenase from soybean; *N,O*-bis-(trimethylsilyl)-trifluoroacetamide with 1-trimethylchlorosilane (BSTFA + TMCS); indomethacin; linoleic acid; ursolic acid; oleanolic acid; β-sitosterol; methyl benzoate; methyl salicylate; and docosane were purchased from Sigma-Aldrich (Seelze, Germany/St. Louis, MO, USA). Heparin sodium salt was purchased from WZF Polfa (Warsaw, Poland). HPLC grade solvents (methanol and orthophosphoric acid) were from Avantor Performance Materials (Avantor PM, Gliwice, Poland). All other chemicals and solvents were of analytical grade and supplied by Avantor PM. In all analyses redistilled water was used. Enzymatic tests were done using 96-well plates and monitored using a Synergy 4 microplate reader (BioTek, Winooski, VT, USA). Column chromatography (CC) was performed on silica gel 60 G (0.2–0.5 mm, Macherey-Nagel, Düren, Germany). Analytical TLC was carried out on silica gel 60 G precoated plates (Merck, Warsaw, Poland) using horizontal DC chambers (Chromdes, Lublin, Poland). Flash chromatography was carried out on an Interchim puriFlash^TM^ 430evo chromatograph (Interchim Inc., San Pedro, CA, USA) equipped with a UV-Vis detector scanning in the wavelength range of 200–600 nm, a fraction collector, and Interchim puriFlash Silica HC (50 µm, 72 mm × 15 mm, 4 g) preparative columns. The flow rate was 7 mL/min. The mobile phase consisted of petroleum ether (A), chloroform (B), and methanol (C). A linear gradient system was used for elution (A:B:C): 0–9 min, 75:25:0 (*v*/*v*/*v*); 9–10 min, 49:49:2; 10–15 min, 45:45:10. Before injection, samples were dissolved in chloroform. Melting points (uncorrected) were determined on a Boetius apparatus (Carl Zeiss, Jena, Germany). UV-Vis spectra, before and after adding ionization and complexation reagents, were recorded at 25 °C on a UV-1601 Spectrophotometer (Rayleigh, Beijing, China) in methanol, according to Mabry et al. [24]. ESI-MS spectra were measured on a UHPLC-3000 RS chromatograph (Dionex, Dreieich, Germany) equipped with a dual low-pressure gradient pump, an autosampler, a diode array detector, and an AmaZon SL ion trap mass spectrometer with an ESI interface (Bruker Daltonik, Bremen, Germany). IR spectra were recorded in KBr on a Bruker Alpha FT-IR spectrometer (Bruker Daltonik). Optical rotation (${\left[\alpha \right]}_{D}^{20}$) was measured in chloroform on a PolAAr 3001 polarimeter (Optical Activity, Ramsey, UK). ^1^H-NMR, ^13^C-NMR, ^1^H-^1^H COSY, HMQC, and HMBC spectra were recorded at 25 °C on a Bruker Daltonik III 600 MHz spectrometer in CDCl_3_ and with TMS as the internal standard.

### 3.2. Plant Material and Preparation of Plant Extracts

#### 3.2.1. Plant Material

Leaves of *Gaultheria procumbens* L. were collected in October 2014 (for preparative extraction, isolation, GC-MS analysis, and activity studies), as well as monthly between April and October 2014 (for analysis of the seasonal variation of triterpene acids) in the nursery-garden of Ericaceae plants, Gospodarstwo Szkolkarskie Jan Cieplucha (54°44′ N, 19°18′ E), Konstantynow Lodzki, Poland, where the plants grew in an open area. The seeds for the nursery were imported from the William J. Beal Botanical Garden (Michigan State University, East Lansing, MI, USA), and authenticated by Piotr Banaszczak, Head of the Arboretum, Forestry Experimental Station of Warsaw University of Life Sciences (SGGW) in Rogow, Poland. The voucher specimen was deposited in the herbarium of the Department of Pharmacognosy, Medical University of Lodz, Poland, with the number KFG/HB/14001-GPRO. Leaf samples for the analysis of seasonal variation were collected each time from ten random plants selected from healthy individuals separated by minimum 5 m and representing the average development stage at the sampling site. Samples of the plant material were air-dried under normal conditions, powdered with an electric grinder, and sieved through a ø 0.315-mm sieve.

#### 3.2.2. Preparation of Dry Lipophilic Leaf Extracts

The preparative sample (1000 g) of the pulverized plant material was extracted in a Soxhlet apparatus first with petroleum ether (1 L, 72 h) and then with chloroform (1 L, 72 h). The obtained fractions were concentrated *in vacuo* to yield petroleum ether (**PE**, 122.0 g, 12.2% DW) and chloroform (**CHE**, 74.0 g, 7.4% DW) dry extracts. The extraction yield was defined as the amount of the dried extract obtained from 100 g of the dried plant material.

#### 3.2.3. Preparation of Plant Extracts for UHPLC-PDA Quantification of Triterpene Acids

The accurately weighed samples of the pulverized plant material (300 mg) were sequentially refluxed twice for 30 min with 30 mL of chloroform, and finally once for 30 min with 30 mL of chloroform–methanol (1:1, *v*/*v*). The combined extracts were evaporated to dryness *in vacuo*. The residue was dissolved in 4 mL of chloroform–methanol (1:1, *v*/*v*), diluted with methanol to 10 mL, filtered through a PTFE syringe filter (25 mm, 0.2 μm, Vitrum, Prague, Czech Republic) and directly analyzed by UHPLC-PDA (Section 3.3.3). The determinations were performed after three separate extractions of each sample.

### 3.3. Phytochemical Profiling

#### 3.3.1. Qualitative GC-MS Analysis of *G. procumbens* Dry Leaf Extracts

The analysis was performed on an Agilent 6890N Network Gas Chromatograph combined with an Agilent 5973 Mass Selective Detector and equipped with a silica fused capillary column DB-5MS (30 m × 0.25 mm i.d., film thickness 0.25 μm; Agilent Technologies, Santa Clara, CA, USA). The oven temperature was programmed from 80 °C to 270 °C at a rate of 10 °C/min. The flow rate of the carrier gas (helium) was 0.6 mL/min. The injector and the interface (between GC and MS) were kept at 250 °C. The samples of the dry extracts (**PE** and **CHE**, 10 mg/mL) and standards (1 mg/mL) were dissolved in toluene and derivatized with BSTFA+TMCS (100 μL) for 3 h in the dark. After this time the samples were injected (1 μL) into the GC-MS system. Identification of the compounds was performed on the basis of their chromatographic behavior (retention times) and MS-spectra in comparison with the standards (both retention times and MS-data) and W9N08.L and NIST 05.L library databases (MS-fragmentation). In all cases when the standards were unavailable, the quality of the library matches ranged from 90% to 99%.

#### 3.3.2. Isolation and Structure Elucidation

The preparative **CHE** sample (70 g) was dissolved in boiling methanol (500 mL). After cooling, the precipitated waxes were removed by filtration. The filtrate was evaporated to dryness *in vacuo* and then re-dissolved in chloroform (50 mL). The purified extract was separated by column chromatography on silica gel using benzene-ethyl acetate with ethyl acetate gradient as an eluent. The TLC chromatograms (silica gel; benzene–chloroform–methanol, 20:30:4, *v*/*v*/*v*) were analyzed in UV light at 365 nm, before and after detection at 110 °C with a Liebermann-Burchard reagent (ethanol–acetic anhydride–96% sulphuric acid, 10:1:1, *v*/*v*/*v*). Based on the chromatographic profile three fractions were selected: T1 (459 mg; eluted with benzene–ethyl acetate, 90:10, *v*/*v*), T2 (1.321 g; benzene–ethyl acetate, 85:15, *v*/*v*) and T3 (458 mg; benzene–ethyl acetate, 50:50, *v*/*v*). After evaporating the solvents, the fractions T1, T2 and T3 were re-dissolved in small amounts of chloroform and separated independently by flash chromatography (Section 3.1) to give three pure compounds: **DL** (55 mg), **DS** (57 mg) and **VO** (82 mg), respectively.

Compound **DL** (yellow needles), m.p. 215–220 °C, positive reaction (orange color) in the Shinoda test. UV (MeOH) λ_max_ nm: 271, 340; NaOMe 255, 269; AlCl_3_ 272, 299 (sh), 349; AlCl_3_-HCl 275, 307 (sh), 356; NaOAc 271, 375; NaOAc-H_3_BO_3_ 271, 340. ESI-MS: *m*/*z* [M − H]^−^ 327 (100%), MS^2^: [M − H − CH_3_]^−^ 312 (91.3%). ^1^H- and ^13^C-NMR data are presented in Table 2.

Compound **DS** (yellow needles), m.p. 285–290 °C, positive reaction (orange color) in the Shinoda test. UV (MeOH) λ_max_ nm: 274, 331; NaOMe 259 (sh), 271, 385; AlCl_3_ 292 (sh), 300, 354; AlCl_3_-HCl 289 (sh), 300, 353; NaOAc 272, 385; NaOAc-H_3_BO_3_ 273, 335. ESI-MS: *m*/*z* [M − H]^−^ 297 (100%), MS^2^: [M − H − CH_3_]^−^ 282 (84.5%). ^1^H and ^13^C-NMR data are presented in Table 2.

Compound **VO** (white needles), m.p. 105–108 °C, positive reaction (yellow color) with Liebermann-Burchard reagent. ${\left[\alpha \right]}_{D}^{20}$ = +211.5° (*c* = 1.06 g/100 mL, CHCl_3_). UV (MeOH) λ_max_ nm: 236. IR ν_max_ KBr cm^−1^: 3355 (OH), 2864, 1659 (C=O), 1431, 1387, 1277, 1019, 970. ESI-MS: *m*/*z* [2M + H]^+^ 449 (100%), MS^2^: [M + H]^+^ 225 (20.7%), [M + H − CH_3_]^+^ 207 (100%), [M + H − 2CH_3_]^+^ 189 (13.8%). ^1^H- and ^13^C-NMR data are presented in Table 3.

#### 3.3.3. Quantitative UHPLC-PDA Analysis of Triterpene Acids in *G. procumbens* Leaves

UHPLC-PDA analyses were carried out on an Agilent Technologies 1290 Infinity chromatograph (Agilent Technologies) according to the method of Owczarek et al. [45]. The system was equipped with a photodiode array detector (PDA), thermostat, and autosampler. Separations were carried out on a Zorbax Eclipse XDB-C18 column (1.8 μm, 100 mm × 3.0 mm I.D.; Agilent Technologies). The mixture 90:10 (*v*/*v*) of methanol and 1% (*w*/*v*) aqueous orthophosphoric acid was used as the mobile phase for isocratic elution. The injection volume was 3 µL. The flow rate was 0.44 mL/min. The temperature was set at 18°C. The detection wavelength was 215 nm. The analytes were identified by comparison on their retention times and UV spectra with that of the reference standards. The standard stock solutions for calibration were prepared in triplicate in methanol and serially diluted (in two replicates) with the same solvent to six concentration levels within the range of approximately 1.5–150.0 µg/mL for **OA** and 3.0–325.0 µg/mL for **UA**. Each extract and standard samples were directly injected into the UHPLC system in triplicate. Results were calculated in mg per g of dry weight of the plant material.

### 3.4. Biological Activity Testing

#### 3.4.1. Hyaluronidase Inhibition Test

Inhibitory activity of the tested analytes (extracts and individual compounds) was determined by turbidimetric method adapted for 96-well microtiter plates as described previously [52]. The protocol was started by adding 20 μL of the tested analyte solution in monosodium phosphate buffer (pH = 7.0) to 40 μL of hyaluronidase solution (22.55 U/mL) in monosodium phosphate buffer (pH = 7.0) and the mixture was incubated at 37.0 ± 0.1 °C in the dark for 10 min. After this time 40 μL of hyaluronic acid solution (0.03%, *w*/*v*) in monosodium phosphate buffer (pH = 5.35) was added and the mixture was further incubated at 37.0 ± 0.1 °C in the dark for 45 min. Finally 300 μL of bovine serum albumin solution (0.1%, *w*/*v*) in sodium acetate buffer (pH = 3.75) was added to the mixture and incubated in room temperature for 10 min. Changes in turbidity were measured at 600 nm. All analytes were tested in the concentration range of 100−1000 μg/mL at minimum five levels to calculate the IC_50_ values. At each level three independent experiments were carried out in duplicate. Heparin was used as a positive control. Activity of the tested analytes was calculated as inhibition percentage (% Inhibition) of hyaluronidase according to the Equation (1):

(1) $$\%\;{}_{\mathrm{Inhibition}}=100\times \left(1-\left(\frac{A_{HA}-A_{AN}}{A_{HA}-A_{HYAL}}\right)\right),$$

where *A_HA_*—absorbance of solution without the enzyme, *A_HYAL_*—absorbance of solution without the tested analyte (negative control), *A_AN_*—absorbance of solution with the tested analyte.

#### 3.4.2. Lipoxygenase Inhibition Test

Inhibitory activity of the tested analytes (extracts and individual compounds) was determined by a spectrophotometric method adjusted to a 96-well microtiter plates according to Granica et al. [53]. The protocol was started by adding 50 μL of the tested analyte solution in sodium borate buffer (pH = 9.0), then 50 μL of linoleic acid solution (134 μM) was added, followed by 50 μL of lipoxygenase solution (167 U/mL) in sodium borate buffer (pH = 9.0). The reaction mixture was vigorously shaken and changes in absorbance were measured at 234 nm over a period of 15 min with 1 min intervals. All analytes were tested in the concentration range of 50−1000 μg/mL at minimum five levels to calculate the IC_50_ values. At each level three independent experiments were carried out in duplicate. Indomethacin was used as a positive control. Activity of the tested analytes was calculated as inhibition percentage (% Inhibition) of lipoxygenase according to Equation (2):

(2) $$\%\;{}_{\mathrm{Inhibition}}=100\times \left(1-\left(\frac{A_{AN}-A_{LA}}{A_{LOX}}\right)\right),$$

where *A_AN_—*absorbance of solution with the tested analyte, *A_LA_*—absorbance of solution without the enzyme, *A_LOX_*—absorbance of solution without the tested analyte (negative control). The absorbance value in the 10th minute of the reaction were used for the calculation.

### 3.5. Statistical and Data Analysis

The results were expressed as means ± standard deviation (SD) of replicate determinations. The statistical analyses (calculation of SD, one-way analysis of variance, HSD Tukey tests and linearity studies) were performed using the Statistica12Pl software for Windows (StatSoft Inc., Krakow, Poland), with *p* values less than 0.05 being regarded as significant.

## 4. Conclusions

This paper is the first report on the phytochemical profile and anti-inflammatory activity of the lipophilic leaf extracts of *G. procumbens* and *Gaultheria* plants in general. The study demonstrated that both petrol and chloroform extracts are rich in various lipophilic compounds. Aliphatic hydrocarbons, alcohols, and carboxylic acids dominated among the waxy substances, and ursolic and oleanolic acids among terpenoids. The simple phenols were mainly represented by methyl salicylate and methyl benzoate. The lipophilic extracts exhibited a relatively high inhibitory activity towards hyaluronidase and moderate anti-lipoxygenase potential compared to the capacity of positive standards (heparin and indomethacin), which indicated their co-responsibility for anti-inflammatory activity of the wintergreen leaves. The final activity of the extracts was arising from additive and synergistic effects of numerous components. Among pure constituents, the most anti-inflammatory active was (6*S*,9*R*)-vomifoliol, but ursolic and oleanolic acids were recommended as analytical markers for standardization of the plant material due to the best activity-concentration relationship. Across the growing season the highest levels of the markers were accumulated between August and October, which are thus recommended as optimal for harvesting of high quality plant material under Polish climate conditions. The chemical profile of the wintergreen lipophilic leaf extracts and existing knowledge of its main components form a basis for wider application of the extracts as preventive agents against inflammation-related disorders. However, further studies would be desirable in order to clarify the toxicity, bioavailability and other biological properties of the extracts and compounds presented here.

## Figures and Tables

**Table 1:** GC-MS data of identified constituents in the *G. procumbens* dry lipophilic leaf extracts.

No.	Compound	R_t_^a^	Molecular		Q ^b^	Relative Content (%)	
			Weight	Formula		PE ^c^	CHE ^d^
**1**	methyl salicylate (**SM**) ^e^	7.84	152	C_8_H_8_O_3_	100	2.31	6.88
**2**	trimethylsilanol ^g^	9.01	90	C_3_H_10_OSi	99	0.86	
**3**	methyl benzoate (**BM**) ^g^	10.53	136	C_8_H_8_O_2_	91	21.59	10.03
**4**	4-hydroxyphenylethanol ^g^	12.07	456	C_8_H_10_O_2_	99		2.78
**5**	*n*-dodecanoic acid (lauric acid) ^g^	12.61	200	C_12_H_24_O_2_	99	0.32	
**6**	*m*-methoxybenzoic acid (*m*-anisic acid) ^g^	13.39	152	C_8_H_8_O_3_	98		3.60
**7**	(6*S*,9*R*)-vomifoliol (**VO**) ^e^	13.72	220	C_13_H_20_O_3_	100		4.35
**8**	neophytadiene ^g^	13.79	278	C_20_H_38_	99	3.44	5.36
**9**	*n*-hexadecanoic acid (palmitic acid) ^g^	14.86	256	C_18_H_36_O_2_	99	1.42	2.19
**10**	phytol ^g^	15.49	296	C_20_H_40_O	98	0.69	
**11**	*n*-octadecanoic acid (stearinic acid) ^g^	15.79	284	C_18_H_36_O_2_	96	0.21	
**12**	pentacosane ^g^	16.86	352	C_25_H_52_	99	0.48	
**13**	heneicosane ^g^	17.25	296	C_21_H_44_	97	0.34	
**14**	*n*-docosanoic acid (behenic acid) ^g^	17.42	340	C_22_H_44_O_2_	94	0.19	
**15**	heptacosane ^g^	17.69	380	C_27_H_56_	99	2.40	
**16**	tetracosan-1-ol ^g^	17.89	354	C_24_H_40_O	98	0.06	
**17**	hexacos-1-ene ^g^	18.14	364	C_26_H_52_	93	2.14	
**18**	13-docosenamide (erucamide) ^g^	18.15	337	C_22_H_43_NO	97		3.43
**19**	*n*-tetracosanoic acid (lignoceric acid) ^g^	18.35	368	C_24_H_48_O_2_	99	0.02	
**20**	squalene ^g^	18.38	410	C_30_H_50_	99	0.53	
**21**	tetracosane ^g^	18.69	278	C_24_H_50_	94		0.38
**22**	octacosane ^g^	18.73	394	C_28_H_58_	99	11.72	
**23**	hexacosan-1-ol ^g^	18.96	382	C_26_H_54_O	90	0.31	
**24**	hexadecane ^g^	19.31	226	C_16_H_34_	95	1.62	
**25**	nonacosane ^g^	20.03	278	C_29_H_60_	96		0.66
**26**	8-demethyllatifolin (**DL**) ^e^	20.12	328	C_18_H_16_O_6_	100		1.13
**27**	docosane (**DOC**) ^g^	20.14	310	C_22_H_46_	100	18.86	
**28**	heptacosan-1-ol ^g^	20.24	396	C_27_H_56_O	94	0.05	
**29**	octacosan-1-ol ^g^	20.39	410	C_28_H_58_O	91	0.28	
**30**	stigmasta-3,5-diene ^g^	20.51	396	C_29_H_48_	90	0.11	
**31**	α-tocopherol ^g^	20.60	430	C_29_H_50_O_2_	99	0.27	
**32**	8-demethylsideroxylin (**DS**) ^e^	20.89	298	C_17_H_14_O_5_	100		2.25
**33**	*n*-hexacosanoic acid (cerotic acid) ^g^	21.27	396	C_26_H_52_O_2_	90	0.18	
**34**	tritriacontane ^g^	21.96	464	C_33_H_68_	98	2.46	
**35**	campesterol ^g^	22.01	400	C_28_H_48_O	97	0.01	
**36**	β-sitosterol (**β-SIT**) ^g^	23.08	414	C_29_H_50_O	100	2.68	
**37**	β-amyrin ^f^	23.35	426	C_30_H_50_O	100	0.98	
**38**	α-amyrin ^f^	23.89	426	C_30_H_50_O	100	3.86	
**39**	oleanolic acid (**OA**) ^e^	26.72	456	C_30_H_48_O_3_	100	1.70	10.11
**40**	ursolic acid (**UA**) ^e^	27.67	456	C_30_H_48_O_3_	100	4.27	28.82
			**Total:**			**86.36**	**81.97**

^a^ R_t_, retention time (min); ^b^ Q, quality of the library matches (%); ^c,d^ relative concentrations of analytes in petrol (**PE**) and chloroform (**CHE**) extracts according to peak area ratio (%) observed in the total ion chromatograms; ^e^ identified with the isolated compounds; ^f^ identified with the corresponding standards; ^g^ identified based on the W9N08.L and NIST 05.L library databases and available literature.

**Table 2:** ^1^H- (600 MHz) and ^13^C-NMR (151 MHz) data for compounds **DL** and **DS**.

Carbon	Compound DL (CDCl_3_)		Compound DS (CDCl_3_)	
	δ_H_ (ppm) ^a^	δ_C_ (ppm) ^a^	δ_H_ (ppm) ^a^	δ_C_ (ppm) ^a^
**2**		155.5		155.7
**3**		139.1	6.58 (1H, s)	101.1
**4**		178.7		177.9
**5**		158.3		157.3
**6**		108.7		108.3
**7**		163.5		163.1
**8**	6.38 (1H, s)	89.1	6.42 (1H, s)	90.2
**9**		155.0		154.6
**10**		105.9		104.8
**1′**		123.4		122.6
**2′, 6′**	7.97 (2H, d, *J* = 8.7 Hz)	130.4	7.74 (2H, d, *J* = 8.7 Hz)	130.2
**3′, 5′**	6.89 (2H, d, *J* = 8.7 Hz)	115.6	6.91 (2H, d, *J* = 8.7 Hz)	115.6
**4′**		157.8		159.3
**11**	2.05 (3H, s)	7.2	2.05 (3H, s)	7.2
**12**	3.79 (3H, s)	60.1	3.86 (3H, s)	59.7
**13**	3.84 (3H, s)	55.9		–

^a^ assignments confirmed by ^1^H-^1^H COSY, HMQC, and HMBC experiments.

**Table 3:** ^1^H- (600 MHz) and ^13^C-NMR (151 MHz) data for compound **VO**.

Carbon	Compound VO (CDCl_3_)	
	δ_H_ (ppm) ^a^	δ_C_ (ppm) ^a^
**1**		41.2
**2a**	2.24 (1H, d, *J* = 16.9 Hz)	49.7
**2b**	2.44 (1H, d, *J* = 16.9 Hz)	
**3**		197.9
**4**	5.90 (1H, br s)	126.9
**5**		162.6
**6**		79.1
**7**	5.79 (1H, dd, *J*_1_ = 0.8 Hz, *J*_2_ = 15.8 Hz)	129.0
**8**	5.85 (1H, dd, *J*_1_ = 5.3 Hz, *J*_2_ = 15.8 Hz)	135.8
**9**	4.40 (1H, dq, *J*_1_ = 5.3 Hz, *J*_2_ = 6.4 Hz)	68.0
**10**	1.30 (3H, d, *J* = 6.4 Hz)	23.8
**11**	1.89 (3H, d, *J* = 1.1 Hz)	18.9
**12**	1.01 (3H, s)	22.9
**13**	1.08 (3H, s)	24.1

^a^ assignments confirmed by ^1^H–^1^H COSY, HMQC, and HMBC experiments.

## Supplementary Materials

Supplementary materials are available online. Figure S1: GC chromatogram of *G. procumbens* petrol extract, Figure S2: GC chromatogram of *G. procumbens* chloroform extract, Figure S3: UHPLC chromatogram of *G. procumbens* triterpene acids fraction, Table S1: GC-MS full data for the *G. procumbens* dry lipophilic leaf extracts.
